# Mesenteric Lymphatic Alterations Observed During DSS Induced Intestinal Inflammation Are Driven in a TLR4-PAMP/DAMP Discriminative Manner

**DOI:** 10.3389/fimmu.2019.00557

**Published:** 2019-03-26

**Authors:** Matthew Stephens, Shan Liao, Pierre-Yves von der Weid

**Affiliations:** ^1^Department of Physiology and Pharmacology, Inflammation Research Network, Snyder Institute for Chronic Diseases, University of Calgary, Calgary, AB, Canada; ^2^Department of Microbiology, Immunology and Infectious Diseases, Inflammation Research Network, Cumming School of Medicine, Snyder Institute for Chronic Diseases, University of Calgary, Calgary, AB, Canada

**Keywords:** lymphatics, mesentery, toll-like receptors, inflammation, lipopolysaccharides, colitis

## Abstract

**Background:** Inflammatory bowel disease (IBD) is characterized by both acute and chronic phase inflammation of the gastro-intestinal (GI) tract that affect a large and growing number of people worldwide with little to no effective treatments. This is in part due to the lack of understanding of the disease pathogenesis and also the currently poorly described involvement of other systems such as the lymphatics. During DSS induced colitis, mice also develop a severe inflammation of terminal ileum with many features similar to IBD. As well as inflammation within the ileum we have previously demonstrated lymphatic remodeling within the mesentery and mesenteric lymph nodes of DSS-treated mice. The lymphatic remodeling includes lymphangiogenesis, lymphatic vessel dilation and leakiness, as well as cellular infiltration into the surrounding tissue and peripheral draining lymph nodes.

**Methods:** Intestinal inflammation was induced in C57BL/6 mice by administration of 2.5% DSS in drinking water for 7 days. Mice were treated with TLR4 blocker C34 or Polymyxin-B (PMXB) daily from days 3 to 7 of DSS treatment via I.P. injection, and their therapeutic effects on disease activity and lymphatic function were examined. TLR activity and subsequent effect on lymphangiogenesis, lymphadenopathy, and mesenteric lymph node cellular composition were assessed.

**Results:** DSS Mice treated with TLR4 inhibitor, C34, had a significantly improved disease phenotype characterized by reduced ileal and colonic insult. The change correlated with significant reduction in colonic and mesenteric inflammation, resolved mesenteric lymphangiectasia, and CD103^+^ DC migration similar to that of healthy control. PMXB treatment however did not resolve inflammation within the colon or associated mesenteric lymphatic dysfunction but did however prevent lymphadenopathy within the MLN through alteration of CCL21 gradients and CD103^+^ DC migration.

**Conclusions:** TLR4 appears to mediate several changes within the mesenteric lymphatics, more specifically it is shown to have different outcomes whether stimulation occurs through pathogen derived factors such as LPS or tissue derived DAMPs, a novel phenomenon.

## Introduction

IBD constitutes of two major phenotypes of gastrointestinal diseases, Ulcerative colitis and Crohn's disease. Both diseases have an inflammatory component which results in impaired nutrient absorption, cell recruitment, and chronic inflammation. During the pathogenesis of IBD, a major alteration occurring within the hosts GI tract is focused upon the lymphatics.

Severe lymphatic remodeling has been observed within the intestinal wall of IBD patients, at the stage of the initial (lacteal) lymphatics, through to the collecting mesenteric lymphatics, and mesenteric lymph nodes (1–5). However, what effect these alterations are having upon disease progression is still not fully understood. The expansion of the lymphatic network, also known as lymphangiogenesis, is mediated through the binding of the lymphatic vascular endothelial selective growth factors VEGF-C and VEGF-D to VEGFR3, and is a common feature in Crohn's disease (6–8). Blockade of lymphangiogenesis through anti-VEGFR3 antibodies do not provide any therapeutic benefit but rather exacerbates submucosal oedema in animal models of IBD (9, 10), while stimulation of lymphatic functions with VEGF-C ameliorates experimental IBD (10). Therefore, it presents the idea that lymphangiogenesis may in fact be a reparative measure in response to inflammation and pro-lymphangiogenic factors, such as VEGF-C, may provide novel strategies for the treatment of chronic inflammatory diseases. Lymphangiectasia, the dilation of lymphatic vessels, is a common sign of collecting lymphatic vessel disruption. It has been shown to be associated in case of inflammation, and intestinal inflammation in particular, with increase permeability of the lymphatics (3, 4). This can result in oedema, hypoproteinaemia, lymphocytopenia, and immunologic anomalies. Another great concern associated with the leakage is lipid absorption issues resulting in weight loss and fat deposition within the mesothelium. To what extent the fat contributes to inflammation, and the effect it has on resident cells, is still not fully elucidated however has been suggested that lymphatic-associated fat can be a source of inflammatory material and may play a greater role in disease pathogenesis than first expected (11–13).

Toll-like receptors (TLRs) play a key role in mucosal innate immunity and may be involved in the pathogenesis of IBD (14). An evolutionarily conserved family of transmembrane pattern recognition receptors, TLRs recognize pathogen-associated molecular patterns (PAMPs) conserved between microbes (15). Activation of TLRs results in the induction of cytokines, chemokines, and antimicrobial molecules, all important factors in the initial innate response aiding in priming the adaptive immune system (16, 17). TLR4 binds the gram-negative bacterial cell wall component lipopolysaccharide (LPS) and through co-receptor MD-2, interaction triggers both MyD88-dependent and independent pathway leading to the translocation of NF-κB and subsequent production of inflammatory cytokines and proteins (18, 19). TLR4-mediated signaling is important for the recruitment of immune cells to the site of inflammation promoting reparative mechanisms, but can be described as a double-edged sword, as aberrant stimulation can induce chronic inflammation (20).

The lymphatic system is a complex network of specialized vessels involved in tissue fluid homeostasis. Lymphatic vessels drain fluid from tissues and associated organs, and propel it unidirectionally as nutrient- and cell-rich “lymph” back into peripheral blood circulation. Initial lymphatic vessels comprised of closed-end, lymphatic capillaries, which branch into tissue then amalgamate to form larger collecting vessels, which, through the presence of smooth muscle cells surrounding the endothelium wall, propel lymph via peristaltic-like contractions toward the draining lymph node. Formation of lymph is believed to occur through the swelling of the interstitium, respiration, arterial pulsations and skeletal movement. Increased interstitial pressure opens the initial lymphatic vessels through small anchoring filaments attaching endothelial cells to the extra-cellular matrix. During inflammatory diseases such as IBD, increased localized swelling within a tissue creates an increased burden upon the draining lymphatics. Within IBD, disruptions in the mesenteric lymphatic architecture has been correlated to worsened disease progression, putting the changes under scrutiny for their potential contribution to pathogenesis (21, 22).

We aimed to determine whether lymphatic disruption in the mouse model of DSS-induced ileitis/colitis, was in portion driven by TLR4. In order to block TLR4 activity directly and indirectly, two drugs were used. The first, Polymyxin-B, inhibits TLR4 recognition of LPS by binding the lipid-A component of LPS preventing recognition by the receptor. The other, C34, is a direct chemical inhibitor of TLR4 binding to the receptor in an antagonistic and competitive manner. Identified by Neal and colleagues, C34 was a potent TLR4 blocker in enterocytes and macrophages *in vitro*, and reduced systemic inflammation in mouse models of endotoxemia and necrotizing enterocolitis (23). These treatments allowed us to differentiate between the activation of TLR4 by LPS or by TLR4-directed agonists of another source.

## Materials and Methods

### Mice

All mice used were housed at constant temperature (22°C) on a 12:12-h light-dark cycle, with food and water *ad libitum*. The animal handling and experiments were approved by the University of Calgary Animal Care and Ethics Committee and conformed to the guidelines established by the Canadian Council on Animal Care.

### Cell Culture

HEK293 (TLR4/MD2/CD14) dual reporter cells (Invivogen, USA Cat. No HKD-mTLR4ni) were maintained in supplemented DMEM (High Glucose) 10% FCS with Normocin, Hygromycin Gold, and Zeocin as per manufacturers instruction. Cells were passaged every 3–5 days at 80% confluency and maintained in a 37°C incubator, 5% CO_2_ atmosphere. All cells were used between passages 3–8 and experiments were performed on 3 or more distinct passages of cells.

### Induction of Colitis and Administration of Treatments

#### Acute DSS

Six-week-old C57BL/6 mice were obtained from Jackson Laboratories. Colitis was induced in these mice by administration of 2.5% (weight/vol) dextran sulfate sodium (DSS; Affymetrix, Cleveland, Ohio, USA) in drinking water for 7 days. Sham mice were given normal drinking water. I.P. injections of C34 (50 mg/kg) (Tocris, USA, Cat. No 5373) or Polymyxin-B (50 mg/kg) (Sigma Aldrich, USA, Cas. No 1405-20-5) were administered from days 3 to 7 diluted to 200 μl total in saline, control mice received saline only. Mice were euthanized by exposure to isoflurane and cervical dislocation.

### Disease Evaluation

In order to assess the severity of DSS-induced inflammation a multi-parameter approach was used in order to quantify inflammation by region. Colon shortening, a common sign of inflammation-driven fibrosis, was measured as a marker of colonic inflammation. Differences in weight were calculated as the percentage weight loss pre- and post-treatment (SHAM/DSS/DSS + treatment). Additionally, fecal matter consistency and visual blood presence were assessed. All of these factors were evaluated, and using the scoring system detailed in **Table 3**, the disease activity score (DAI) was calculated (see Table 1) [adapted from (24)].

**Table 1:** Disease activity index scoring.

**Symptom/score**	**Characteristic**
**BODY WEIGHT LOSS**	
0	No negative change in weight
1	1–5% loss of body weight
2	5–10% loss of body weight
3	10–20% loss of body weight
4	>20% loss of body weight
**STOOL CONSISTENCY**	
0	Normal
1	Loose consistency
2	Watery
3	Slimy diarrhea
4	Severe diarrhea
**BLOOD PRESENCE IN STOOL**	
0	No blood
2	Red feces
4	Visible bleeding

### Alterations in Lymphatics

#### Lymphangiectasia

Lymphatic vessels were identified as initials by positive staining with CCL21 (R&D systems, USA, Cat. No AF457) and as collectors by staining with αSMA (Sigma-Aldrich, USA, Cat. No C6198) in whole-mount mesenteric preparations. Vessel diameters were measured in 3–5 vessels per sample at 3 random sites along each vessel.

#### Lymphadenopathy

Mesenteric lymph nodes (MLNs) were isolated from the mouse, cleaned of fat and connective tissue and measured for both size (lengthways) and weight.

#### Lymphangiogenesis

Lymphangiogenesis was measured via CCL21 staining of mesenteric whole-mounts. Vessel branch points and numbers were determined in a fixed area of interest kept uniform between samples.

### Whole Mount Immunofluorescence

Whole mount mesenteries were fixed on sylgard coated dishes and fixed with 4% PFA for 1 h at room temperature. Tissues were washed, permeabilized in PBST (PBS + 0.03% Triton X-100) and blocked with 2–3% BSA in PBST. Primary antibodies incubation occurred for 24 h at 4°C. Samples were washed three times in PBST (PBS + 0.01% Triton X-100) for 10 minutes per wash and then incubated with secondary antibodies (in 2% BSA containing 0.01% Triton X-100) for 1–2 h. Samples were washed as previously described before preparation for optical clearing. Fat-clearing was obtained by serial ethanol dehydration followed by methyl salicylate (MeS) immersion for 15 min. Immediately after clearing, samples were mounted with DAPI containing mounting medium and installed with a coverslip for imaging. The imaging occurred within 2 h of clearing due to fluorescent diminishment. Vessel diameter and branching was quantified using the LASX software attached to a Leica SP8 confocal microscope.

### Quantitative Real-Time Polymerase Chain Reaction (qPCR)

The total RNA isolated from given samples was purified using the QIAGEN RNA total cleanup kits as per manufacturers instruction. One hundred nanogram of the RNA was converted using EvaGreen RT conversion kit in a gradient thermocycler as per manufacturers description. One nanogram per microliter of the converted cDNA samples was added to EvaGreen SYBR qPCR master-mix and qPCR analysis was performed in an ABI StepOne Plus PCR system. Annealing temperatures were kept at 60°C, and 40 cycles of amplification were performed to produce a sufficient read. Sequences of primers used are detailed in Table 2.

**Table 2:** RT-PCR primer sequences.

**Gene**	**Sense primer (5^**′**^-3^**′**^)**	**Antisense primer (5^**′**^-3^**′**^)**	**Product size (bp)**	**References**
TLR2	AAGAGGAAGCCCAAGAAAGC	CGATGGAATCGATGATGTTG	199	(25)
TLR3	CACAGGCTGAGCAGTTTGAA	TTTCGGCTTCTTTTGATGCT	190	(25)
TLR4	ACCTGGCTGGTTTACACGTC	CTGCCAGAGACATTGCAGAA	201	(25)
TLR5	AAGTTCCGGGGAATCTGTTT	GCATAGCTGAGCCTGTTTC	201	(25)
TLR7	AATCCACAGGCTCACCCATA	CAGGTACCAAGGGATGTCCT	142	(25)
TLR8	GACATGGCCCCTAATTTCCT	GACCCAGAAGTCCTCATGGA	195	(25)
TLR9	ACTGAGCACCCCTGCTTCTA	AGATTAGTAGCGGCAGGAA	198	(25)
VEGFR3	TCTGCTACAGCTTCCAGGTGG	GCAGCCAGGTCTCTGTGGAT	200	(26)
VEGFC	TGTGCTTCTTGTCTCTGGCG	CCTTCAAAAGCCTTGACCTCG	148	N/A
CCL21	GGTTCTGGCCTTTGGCATC	AGGCAACAGTCCTGAGCCC	262	(27)
GAPDH	CTCATGACCACAGTCCATGC	CACATTGGGGGTAGGAACAC	201	(25)

*qPCR primer sets used for the amplification of genes of interest*.

### Total LPS Isolation From Murine Feces

Samples were homogenized in PBS and subsequently filtered in order to remove non-soluble components. Protein content of the fecal homogenate was determined through the Precision Red Protein Quantification assay (Cytoskeleton, Inc., USA Cat. No ADV02). The concentration of samples was then equilibrated to 1 mg/ml through addition of supplemented DMEM, before being assayed for endotoxin content using the chromogenic-Limulus amebocyte lysate (LAL) assay (ThermoFisher, USA, Cat. No 88282). Equal volumes (3.5 ml) of quantified fecal homogenate was then passed through a high capacity endotoxin removal spin column (Pierce, USA. Cat No 88274) reducing LPS content from an average of 87.67 EU/mg/ml (±7.36) to <1.71 EU/mg/ml (±0.39). The bound LPS was removed for later studies.

### HEK-TLR4 Cell Stimulation

Cells were maintained in Normocin, Hygromycin Gold, Zeocin DMEM-high glucose media as per manufacturers instruction. During stimulation Hygromycin Gold and Zeocin were not present in the media. “Endotoxin-low” samples isolated using the PMXB columns were added to HEK293 (TLR4/MD2/CD14) dual reporter cells (Invivogen, USA Cat. No HKD-mTLR4ni) in order to assess the stimulatory capacity of the material through TLR4. Samples were diluted 1:10 in supplemented DMEM to remove toxic effects of salt exchange which occurred during the LPS removal process reducing endotoxin levels to below threshold (<0.1EU/ml) a value at which comparable LPS concentrations do not activate the cells.

### Statistics

Data are expressed as the mean ± one standard error of the mean (SEM). Sample size varies from 3 to 9 as indicated, performed as a minimum experimental triplicate. Statistical significance was assessed through the use of two-tailed unpaired Student's *t*-test for parametric data, while the Mann-Whitney test was performed for non-parametric data. Multiple analyses were performed using a one-way Anova with *post-hoc* Tukey test where indicated. ^*^P < 0.05, ^**^P < 0.01, ^***^P < 0.001, ^****^P < 0.0001.

## Results

### DSS Alters TLR mRNA Expression Within the Mesentery

Previous findings have demonstrated DSS directly impacting on the expression of TLRs within the inflamed colon mimicking that found in the patient cohort (28–31). We wanted to ascertain whether there the same was true for the murine mesentery. In order to do so, qPCR screening of all known murine TLRs, was assessed. Total RNA from the mesentery of sham-control mice, DSS, and other treatments were isolated and converted to cDNA before analysis via SYBR-green qPCR. Of the 14-known murine TLRs only 7 were detectable within the murine mesentery samples (Table 3). Analysis of expression within sham controls provided a baseline for subsequent comparison with DSS which demonstrated a significant upregulation in TLR4 (*P* < 0.0001). We hypothesized that TLR4, which recognize the bacterial component LPS, could be involved heavily in lymphatic-driven inflammation and dysfunction during DSS induced colitis. Additionally, during the progression of DSS induced colitis, the epithelium of the gut is severely disrupted allowing a vast influx of lumenally-derived microbial content into the mesenteric lymphatics.

**Table 3:** TLR4 mRNA expression changes in murine mesentery during DSS treatment.

	**Fold change sham DSS**	***P*-value**
TLR2	2.359 ± 1.313	>0.9999
TLR3	0.3314 ± 0.185	>0.9999
TLR4	221.7 ± 80.93	<0.0001
TLR5	99.15 ± 24.27	0.0324
TLR7	2.565 ± 0.6062	>0.9999
TLR8	5.123 ± 1.245	>0.9999
TLR9	4.959 ± 1.44	>0.9999

*DSS induces expression of TLR4 within the mesentery. Mesenteric lymphatic samples from SHAM and DSS treated mice (2.5% 7d) were analyzed for TLR expression using qPCR. Unpaired Mann-Whitney test, two-tailed*.

### DAMPs Created Within the Colon During DSS Treatment Activate Cells in a TLR4 Dependent Manner

**Figure 1:**
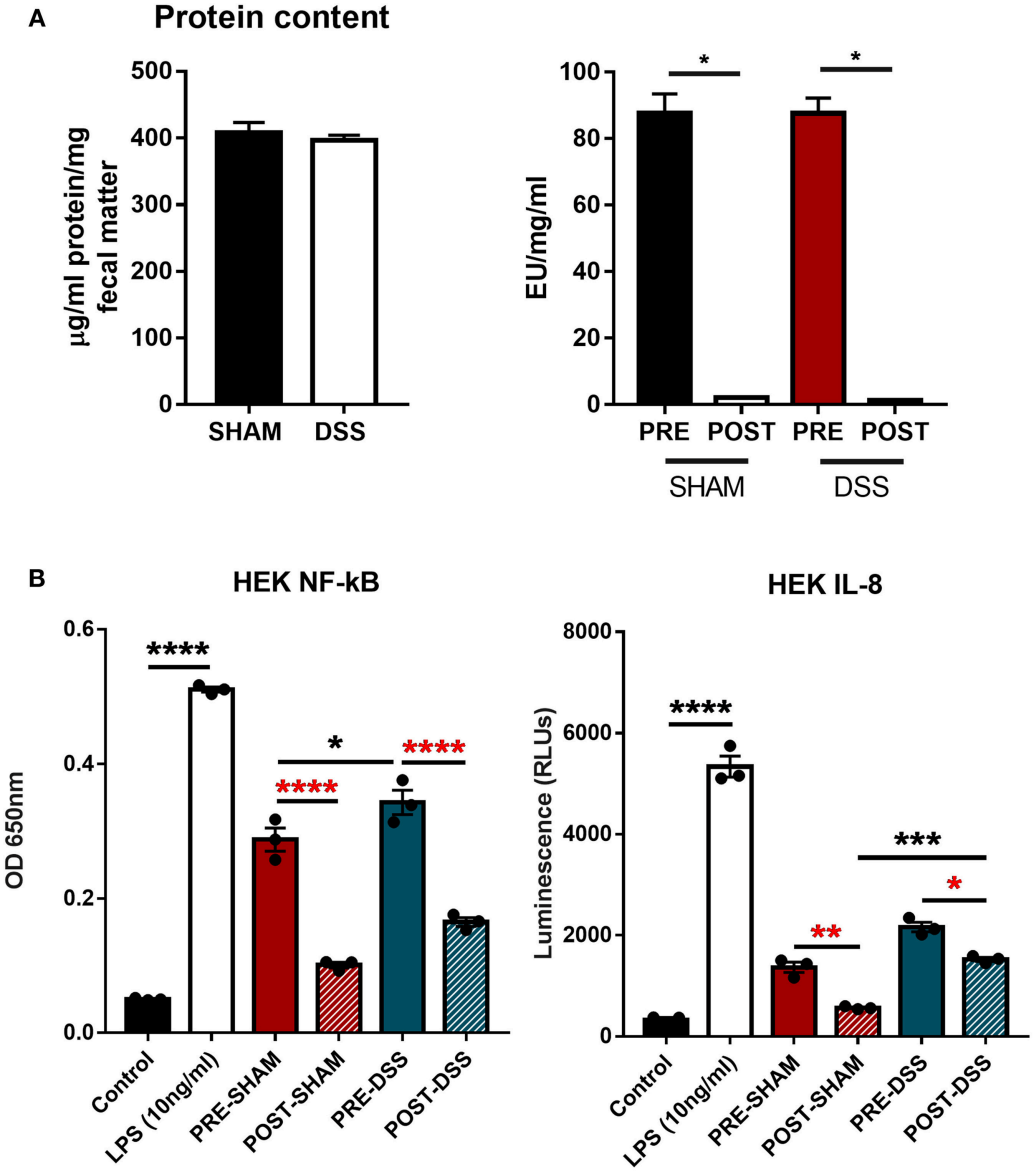
TLR4 activation *in vitro* is triggered by a ligand other than LPS. **(A)** Fecal matter samples from sham and DSS (2.5% 7d) mice were isolated, normalized by protein content, and treated in an endotoxin removal column for 72 h at 4°C (post-sham and post-DSS samples). **(B)** Percentage NF-κB and IL-8 response from samples removed of endotoxin displayed DSS treated mice having a large proportion of activation due to non-LPS derived products. Data is represented as the mean ±SEM of 3 experimental replicates. EU, endotoxin unit. Two-tailed Student's *t*-test and one-way Anova (Tukey *post-hoc* test) and were used in A and B, respectively. *P < 0.05, ***P < 0.001, and ****P < 0.0001.

In order to discern what, within the GI tract, could be activating upon TLR4 directly, samples of colonic fecal matter were collected from sham and DSS treated mice for analysis. Data shown in Figure 1A show protein normalized samples and their subsequent LAL-determined endotoxin levels per milligram of fecal matter from both sham and DSS treated mice. The levels of endotoxin present in the sample are denoted as: PRE (before) and POST (after) endotoxin removal via the Polymyxin-B column. Figure 1B demonstrates that the induction of the NF-κBSEAP (Secreted Embryonic Alkaline Phosphatase) and IL-8 luciferin reporters found within the HEK-TLR4 reporter cells can be driven by substances within the murine fecal matter. A significant reduction in the induction of gene expression can be seen through the removal of LPS. However, a proportion of the (POST) DSS sample can still induce both NF-κB and IL-8 gene expression suggesting other molecules are being recognized by TLR4.

### TLR4 Blockade Through C34 Treatment Ameliorates the Progression of DSS Induced Disease Activity

Being home to the majority of the microbiome, the gut must function effectively as a barrier in order to prevent the influx of microbial pathogens into the normally sterile sub-mucosa. The multi-layered composition of the intestinal tract aids in this function through the secretion of mucins, the epithelial barrier itself and the rapid response of immune cells a site of breach (32). During DSS induced colitis, the breakdown of the epithelial barrier leaves the potential for invasion of commensal bacterium, fungi, viruses and dietary substances to permeate the pseudo-sterile barrier (33, 34). Activation of the resident macrophages, dendritic cells and mast cells within the epithelial sub-lining promotes the recruitment of neutrophils, the induction of pro-reparative measures, and the clearance of antigens to the lymph node in an attempt to create and effective immune response to the infection (35, 36).

**Figure 2:**
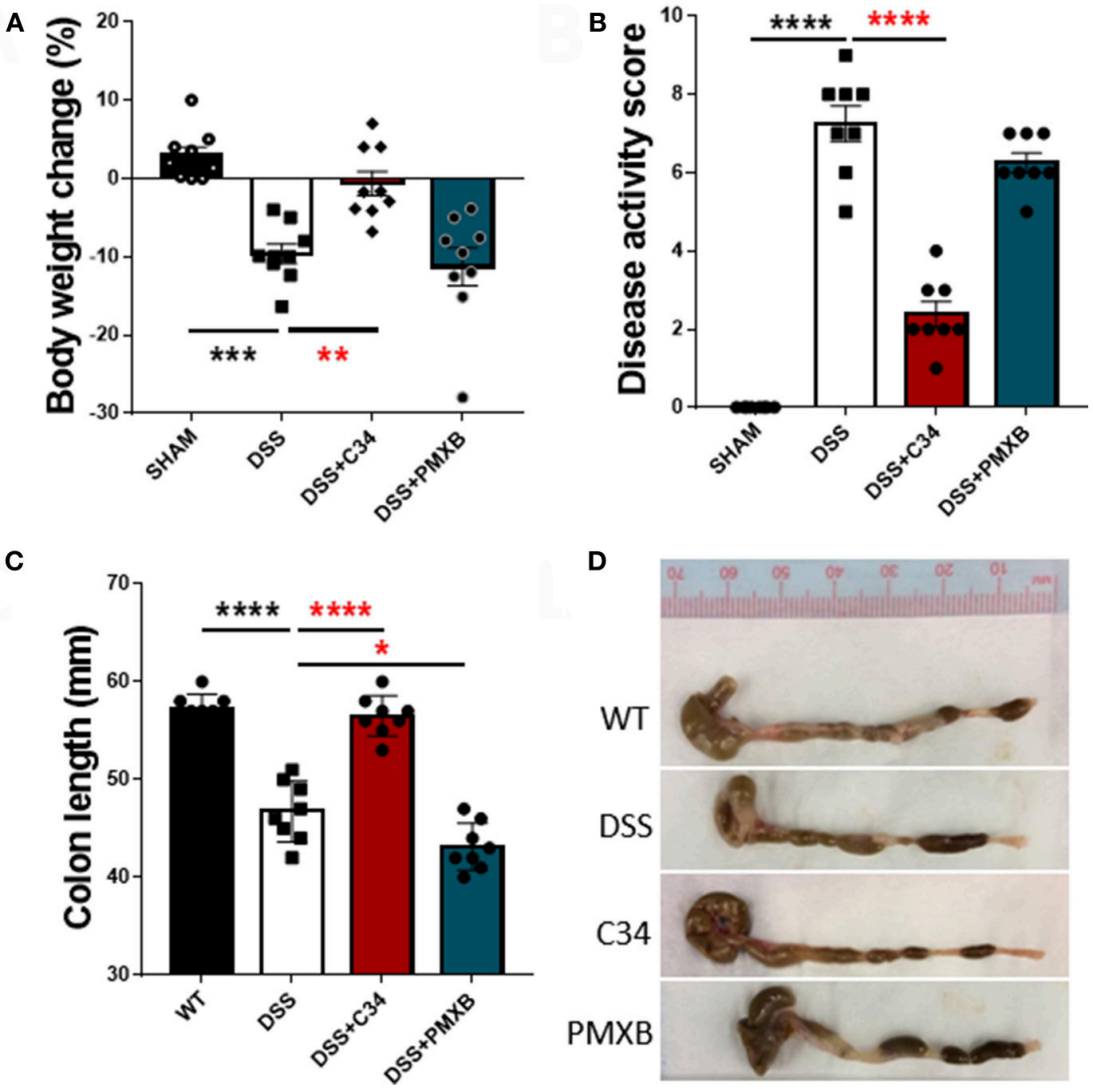
Total TLR4 blockade within the peritoneum ameliorates DSS induced colitis *in vivo*. Mice were treated with DSS 2.5% and additionally with either C34 (50 mg/kg) or PMXB (50 mg/kg) from days 3 to 7. Percent body weight change **(A)**, clinical disease activity score **(B)** and colon length **(C, D)** were measured as macroscopic scores for disease response measures. Data are expressed as the average ±SEM of 3 independent experiments (*n* = 8 in each group). Images are representatives from those experiments. Statistics analyzed using one-way Anova (Tukey *post-hoc* test), *P < 0.05, **P < 0.01, ***P < 0.001, and ****P < 0.0001.

With high levels of bacterial LPS and DAMPs present within the intestinal luminal space, a potential to activate a TLR4 mediated innate immune response is rife. Inflammatory molecules induced by TLR4 activation are documented to negatively impact on lymphatic function, thus potentially reducing flow of antigens to the lymph node and subsequent immunosuppression (37). Therefore, we attempted to determine the effect of inhibition of TLR4 and subsequent effect on inflammation within the local drainage lymphatic system. Mice treated with 2.5% DSS for 7 days, received a daily I.P injection of either saline, C34 or PMXB from days 3 to 7 (See Methods). DSS colitis in mice is characterized by the development of diarrhea, colonic inflammation, and subsequent weight loss. Fecal consistency, blood presence in feces and the extent of the colon shortening was converted to a DAI and recorded as described in Methods (see Table 3). When compared to sham controls, treatment with C34 significantly reduced weight loss (Figure 2A), reduced disease activity score (Figure 2B) and reduced colon shortening (Figure 2C). However, treatment with PMXB did not aid significantly in the characteristic disease phenotype. We also tested an alternative drug delivery method via oral gavage of the treatments in the same dosage and time frame, however, neither treatment alleviated any tested condition (Data not shown).

### TLR4 Modulates Lymphatic Alterations Within the Mesentery in an LPS-Independent Manner

The promotion of lymphangiogenesis within the mesentery of DSS treated animals is a well-documented phenomenon (3, 10). Alongside lymphangiogenesis, vessel dilation mediated by iNOS-dependent production of nitric oxide is a common feature associated with inflammation within the tissue surrounding the lymphatics (37). The dilation often correlates with increased vessel permeability and particulate exchange, although the effectors regulating this process are still unknown. This “leakiness” can disrupt the flow of antigen-bearing immune cells to the lymph node and cause lymph and its content to spill out into the surrounding tissue. We hypothesized that one of the functions of mesenteric lymphatic network expansion is to resorb this lost material.

**Figure 3:**
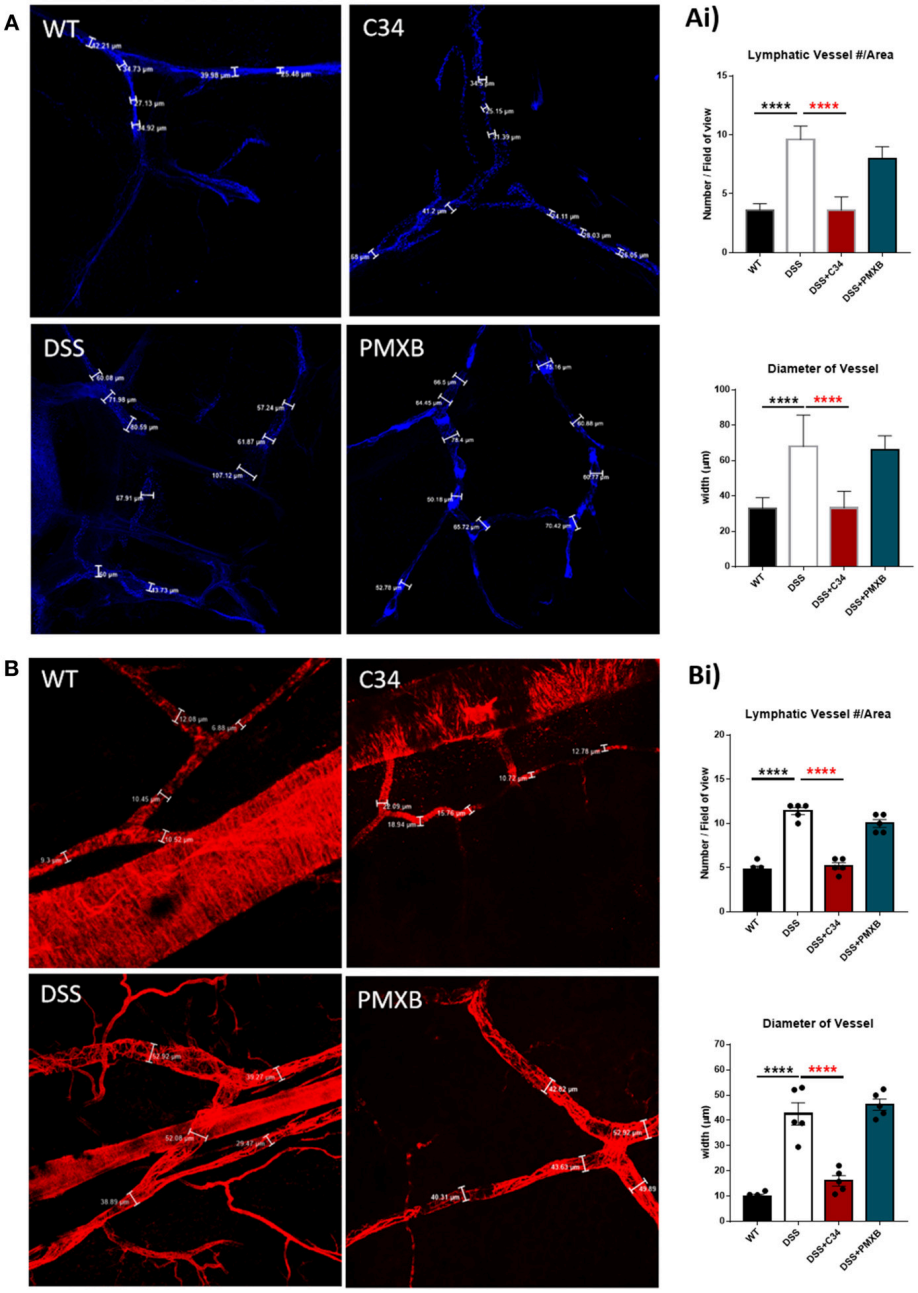
TLR4 blockade ameliorates lymphatic alterations within the mesentery of DSS treated mice. Mice treated with C34 TLR4 total inhibitor reduced lymphangiogenesis and lymphangiectasia within the mesentery however, PMXB treated mice had no significant reduction. Images are representative staining of **(A)** CCL21 positive mesenteric initial lymphatics and **(B)** αSMA positive mesenteric collecting lymphatics, *n* = 3 for each group. Measurements were taken at 3 random points along the vessel width and averaged for each mouse. Branching points were identified and calculated per field of view. Data are expressed as mean ±SEM of 2–3 separate experiments. One-way ANOVA with Tukey *post-hoc* test *P < 0.05, **P < 0.01, ***P < 0.001, and ****P < 0.0001.

Mesenteric sections were isolated from sham and DSS mice treated or not with C34 or PMXB, fixed as whole-mount and stained with lymphatic vessel markers. The initial-lymphatic endothelial marker CCL21, and collecting lymphatic marker αSMA, were used to highlight the border of the lymphatic endothelium and/or smooth muscle layer, allowing vessel size measurement (Figure 3). As illustrated in Figure 3, sham-control collecting lymphatic vessels average luminal diameter and branch points were assessed creating baseline values. DSS treated samples showed extensive expansion of the lymphatic network and a significant increase in lymphatic vessel diameter. Restoration of the normal phenotype was successfully achieved through administration with C34. However, PMXB had no significant effect. This finding was compounded through the analysis of CCL21 mRNA levels within the affected tissue, whereby C34 reduced expression to that of sham control whilst PMXB had no effect on transcript levels. Altogether, these data show mesenteric lymphangiogenesis and lymphangiectasia can be resolved through the I.P. blockade of TLR4 using C34.

### TLR4 Activation Modulates Lymphangiogenic and Inflammatory Molecules Within the Mesentery

**Figure 4:**
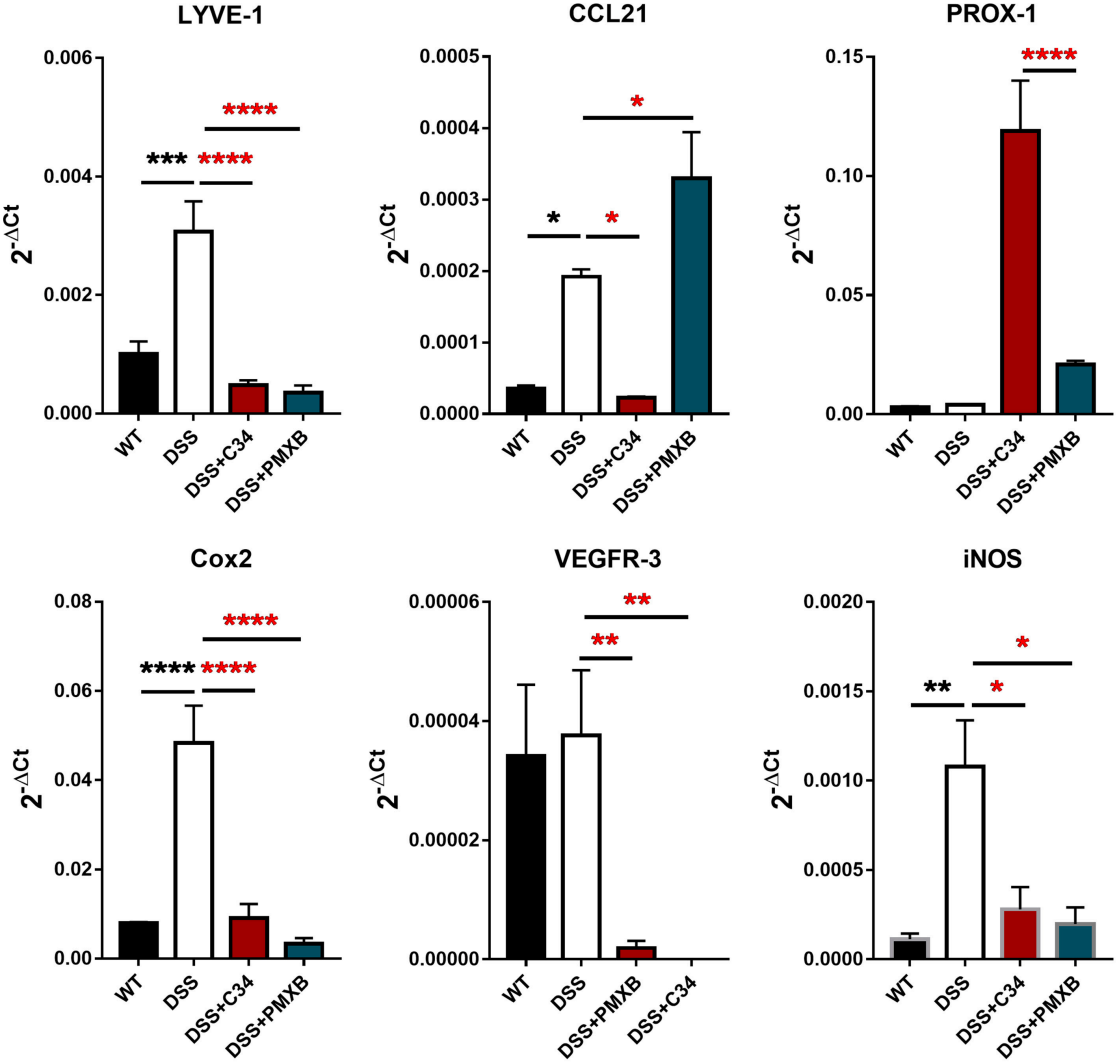
DSS alters lymphatic and inflammatory modulators within the mesentery and is altered through TLR4 blockade. mRNA induction of lymphatic markers LYVE-1, CCL21, Prox-1, VEGFR3 and inflammatory markers COX2 and iNOS were measured on total extractions of mesenteric preparations from SHAM, DSS treated, and DSS + treatment groups. GAPDH was used for normalization to a housekeeping gene and values are expressed as such. Data is mean ±SEM from 5 individuals from 3 separate experiments. One-way ANOVA with Tukey-*post-hoc* test *P < 0.05, **P < 0.01, ***P < 0.001, and ****P < 0.0001.

The inflammation associated with DSS-induced intestinal inflammation promotes lymphangiogenesis within the mesentery as well as altering lymphatic structure through dilation of the collecting lymphatic vessels (Figure 2). Analysis of mRNA levels of poignant lymphatic markers (Figure 4) revealed significant increases in LYVE-1 (*P* < 0.001) and CCL21 (*P* < 0.05) transcription during DSS treatment with the effect ameliorated by treatment with C34. Interestingly, the widely accepted universal lymphatic endothelial marker PROX-1, was not significantly induced through DSS treatment. Rather, inhibition of TLR4 signaling through C34 treatment induced PROX-1 transcription suggesting that TLR4 regulates the lymphangiogenic transcription factor in an activation dependent manner. Additionally, COX2 (*P* < 0.0001) and iNOS (*P* < 0.01) mRNA levels spiked during DSS treatment, indicating signs of mesenteric dysfunction, with both treatments ameliorating this induction suggesting an anti-inflammatory effect of TLR4 inhibition within the mesentery itself. Interestingly VEGFR3 expression was not significantly increased in the DSS group compared to sham but was rather significantly reduced through C34 (*P* < 0.01) and PMXB (*P* < 0.01). Furthermore, analysis of common inflammatory markers (TNFα, IL-1β and IL-6) in paired mesenteric samples showed no significant induction within any group at day 7, inferring the passage of the acute inflammatory phase within that region (Data not shown).

### LPS Drives Lymph Node Expansion and Cellular Migration During DSS Treatment

Lymphadenopathy, or swelling on the lymph node, is a common occurrence during the response to infection presenting in either a localized (regional) or diffuse (generalized) phenotype (38). Immune cells within the mesentery can become activated and promote expression of chemotactic agents within the collecting lymphatics such as CCL21 which, though production of a gradient, attracts CCR7^+^ cells, such as dendritic cells, from the lamina propria to the mesenteric lymph node for antigen presentation (39, 40). These cells accumulate within the lymph node after trafficking antigens from a peripheral site of inflammation, and subsequently produce a wide array of proliferative and chemoattractant agents, which, result in structural remodeling of the node (41, 42). TLR4 activation is known to induce TNF-a production, IL-8 secretion, and matrix protease secretion from a wide variety of cells, including fibroblastic reticular cells and macrophages, key players in lymph node remodeling (43, 44).

**Figure 5:**
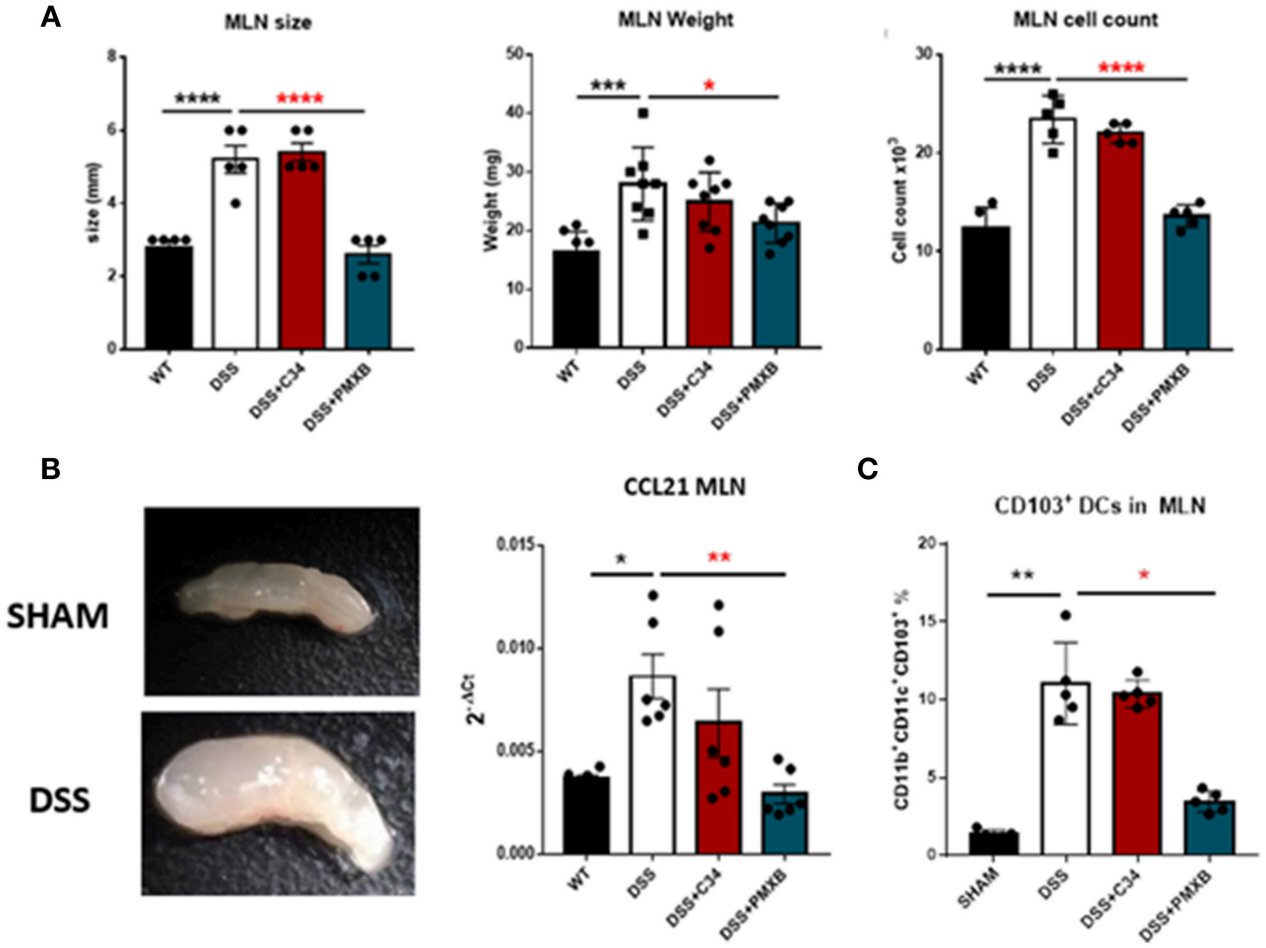
LPS drives lymphadenopathy during DSS treatment augmenting CD103^+^ DC recruitment to the Mesenteric lymph node. **(A)** Isolated MLNs were measured for size, weight and cellularity. **(B)** mRNA expression of CCL21 within the MLN and mesentery during treatments. **(C)** MLN accumulation of CD11c^+^CD11b^+^CD103^+^ Dendritic cells were determined through flow cytometric analysis and quantified within the lymph node in each condition. Data are mean ±SEM of 3 experimental replicates (*n* = 5–8). One-way Anova with Tukey *post-hoc* tests were performed as necessary. *P < 0.05, **P < 0.01, ***P < 0.001, and ****P < 0.0001.

Examination of the lymph node corroborated that DSS had a distinct effect on lymphadenopathy significantly increasing the MLN size (*P* < 0.0001), weight (*P* < 0.001), and cellular content (*P* < 0.0001) (Figure 5A). This effect was not abrogated through C34 blockade of TLR4, however, PMXB treatment reduced significantly the MLN size (*P* < 0.0001), weight (*P* < 0.0001), and cell count (*P* < 0.0001) suggesting the possible involvement of a non-TLR4 dependent LPS interaction in lymphadenopathy. Altogether, these results suggest that TLR4 does not directly influence lymphadenopathy in a DSS model of colonic inflammation but LPS does, and it does so in a TLR4-independent manner. DSS treatment also significantly upregulated CCL21 expression within the MLN (*P* < 0.05) (Figure 5B). C34 in combination with DSS treatment had no effect on CCL21 expression in the MLN, however PMXB treatment significantly reduced it (*P* < 0.01), impacting the recruitment of CCR7^+^ CD103^+^ DCs accumulation (*P* < 0.05) (Figure 5C).

## Discussion

Within the intestine there is a delicate balance between innate immune activation and inflammation. Invasive pathogens can be recognized by a myriad of pattern recognition receptors and induce an inflammatory response, a feature critical to the successful clearance of aforementioned pathogen (45). However, in a system that has the potential to be exposed to an enormous volume of these microbes, a correct magnitude of response is paramount (46). Patients suffering from IBD have an exacerbated and possibly unregulated inflammation within the intestinal tract, a feature that does not resolve normally as expected, but rather perpetuates in a chronic fashion. During the progression of IBD, and perhaps its conception, the structure of the gastrointestinal-associated and mesenteric lymphatic vascular environment changes drastically (22, 47–49). Whether this phenomenon is a direct cause of IBD or a causal agent of IBD is debated. However, from our own lympho-centric opinion, the lymphatic dysfunction itself could cause both. Increased intestinal permeability, combined with reduced lymphatic function could lead to a stagnation of material within the effected intestinal region leading to a hot-spot of inflammation. In the DSS model, intestinal permeability is increased through the chemical ablation of the intestinal epithelium via the formation of nano-lipocomplexes between medium-chain-length fatty acids and DSS, highly abundant in the colon, therefore greatly isolating its effector venue (50). Loss of this barrier allows a vast milieu of microbial and dietary content to enter the submucosa and be directly exposed to the lymphatic system via the initial lacteal vessels. Within this transport system the exposure of immune and stromal cells to bacterial products such as LPS cause a large inflammatory response, tissue remodeling and cellular proliferation/recruitment, a phenomenon well-documented in the lung (51, 52). During the progression of IBD extra-cellular matrix remodeling is common, the role of this remodeling however, is poorly understood and it is thought that extracellular molecules produced during this destructive/reparative stage may in fact perpetuate inflammation (53). We have previously demonstrated that even after the removal of DSS, remodeled lymphatics persist, a phenomenon that is evident with the IBD patient population (3).

TLR4 has been implicated in the pathogenesis of many inflammatory diseases including IBD, as through the recognition of LPS and a wide array of previously mentioned PAMPs and DAMPs, a large inflammatory stimulus can be generated (54). Our data reveals not only the TLR4-driven lymphatic alterations during DSS-induced colitis, but the gross-mechanisms which they act. Through the use of a competitive TLR4 antagonist (C34) and Polymyxin we were able to selectively differentiate between total TLR4-driven lymphatic alterations/consequences during DSS induced colitis, and those driven by LPS. Data presented, indicates that substances separate from LPS, i.e., TLR4-DAMPs, and other PRR PAMPs, modulate inflammation and lymphatics to a much greater extent than previously estimated. This hypothesis was confirmed through the detection and analysis of TLR4 activating material within the colon of DSS mice separate from the LPS content. Currently, specific DAMPs have not been elucidated in this system, however it would be feasible to expect well-published TLR4 DAMPs such as Tenacin-C, HMGB1, HSP90, or S100 proteins to be candidates as levels are known to drastically rise during tissue damage (55–57).

A key marker of correct lymphatic response was the potent lymphadenopathy seen during DSS treatment. This effect was disrupted through the PMXB treatment, accredited to the lack of CD103^+^ DC migration to the lymph node. However, through C34 inhibition of TLR4, CCL21 (a potent lymphatic chemokine) was downregulated in the mesentery but not the lymph node, creating a gradient for increased movement of CCR7^+^ CD103^+^ DCs. This gives partial explanation to the reduced cellular content of the lymph node and significant reduction in lymphadenopathy which was ameliorated through PMXB treatment where the CCL21 content in the lymph node is downregulated. Therefore, any CCL21-driven chemotaxis within the mesentery has no directionality. This also suggests that the lymphadenopathy, whilst caused by LPS, is not driven through TLR4, a novel finding in this context.

We also present evidence supporting our hypothesis that restoration of lymphatic function to a “normal” phenotype, significantly aids in the reparation of DSS-induced disease activity. Achieved through TLR4 blockade via C34, mesenteric lymphatic disruption was significantly reduced, evidenced by reduced lymphangiogenesis and lymphangiectasia. We note that this finding, of reduced lymphangiogenesis improving DSS-induced phenotype, is in somewhat opposition to D'Alessio's, work whereby lymphangiogenesis was beneficial to their model of IBD (10). This discrepancy could be solely contributed to the timing of the treatments or the target itself. Our experimental method and timings were designed to modulate inflammation after its genesis rather than in a preventative capacity. We attempted to modulate TLR4-associated inflammation and therefore the subsequent lymphatic remodeling, whereas D'Alessio and colleagues focused intentionally on promoting lymphatic remodeling through the overexpression of VEGFC, a method that likely had many targets separate from VEGFR3 induced lymphangiogenesis.

We know with certainty that alterations occur within the lymphatics of patients with of IBD and we are able to mimic them in murine models of DSS-induced intestinal inflammation. However, what is not yet understood is whether TLR4 could be a potential target for IBD in humans. Targeting such an important receptor undoubtedly has its risks but data presented in this paper suggest the plasticity of the receptor in delineating pathogenic material from self, a phenomenon that could be utilized in the future for the development of novel treatment of IBD.

## Data Availability

The datasets generated for this study are available on request to the corresponding author.

## Author Contributions

Project planning and experimentation was solely carried out by MS. Interpretation of data, technical advice, and manuscript proofreading was performed by P-YvdW and SL.

### Conflict of Interest Statement

The authors declare that the research was conducted in the absence of any commercial or financial relationships that could be construed as a potential conflict of interest.

## References

[1] Kaiserling E Krober S Geleff S. Lymphatic vessels in the colonic mucosa in ulcerative colitis. Lymphology. (2003) 36:52–61. 12926829

[2] Wen J Tang Q Wu J Wang Y Cai W. Primary intestinal lymphangiectasia: four case reports and a review of the literature. Dig Dis Sci. (2010) 55:3466–72. 10.1007/s10620-010-1161-1 20198428

[3] Rehal S Stephens M Roizes S Liao S von der Weid P-Y. Acute small intestinal inflammation results in persistent lymphatic alterations. Am J Physiol Gastrointest Liver Physiol. (2017) 314:G408–17. 10.1152/ajpgi.00340.2017 29351397

[4] Rehal S von der Weid PY. TNFDeltaARE mice display abnormal lymphatics and develop tertiary lymphoid organs in the mesentery. Am J Pathol. (2017) 187:798–807. 10.1016/j.ajpath.2016.12.007 28183530 PMC5397719

[5] Kovi J Duong HD Hoang CT. Ultrastructure of intestinal lymphatics in Crohn's disease. Am J Clin Pathol. (1981) 76:385–94. 6117198 10.1093/ajcp/76.4.385

[6] Pedica F Ligorio C Tonelli P Bartolini S Baccarini P. Lymphangiogenesis in Crohn's disease: an immunohistochemical study using monoclonal antibody D2–40. Virchows Arch. (2008) 452:57–63. 10.1007/s00428-007-0540-2 18040712

[7] von der Weid PY Rehal S Ferraz JG. Role of the lymphatic system in the pathogenesis of Crohn's disease. Curr Opin Gastroenterol. (2011) 27:335–41. 10.1097/MOG.0b013e3283476e8f 21543977

[8] Rahier JF De Beauce S Dubuquoy L Erdual E Colombel JF Jouret-Mourin A. Increased lymphatic vessel density and lymphangiogenesis in inflammatory bowel disease. Aliment Pharmacol Ther. (2011) 34:533–43. 10.1111/j.1365-2036.2011.04759.x 21736598

[9] Jurisic G Sundberg JP Detmar M. Blockade of VEGF receptor-3 aggravates inflammatory bowel disease and lymphatic vessel enlargement. Inflamm Bowel Dis. (2013) 19:1983–9. 10.1097/MIB.0b013e31829292f7 23835443 PMC3732464

[10] D'Alessio S Correale C Tacconi C Gandelli A Pietrogrande G Vetrano S. VEGF-C–dependent stimulation of lymphatic function ameliorates experimental inflammatory bowel disease. J Clin Invest. (2014) 124:3863–78. 10.1172/JCI72189 25105363 PMC4151217

[11] Gewirtz AT. Deciphering the role of mesenteric fat in inflammatory bowel disease. Cell Mol Gastroenterol Hepatol. (2015) 1:352–3. 10.1016/j.jcmgh.2015.05.004 28210686 PMC5301283

[12] Peyrin-Biroulet L Gonzalez F Dubuquoy L Rousseaux C Dubuquoy C Decourcelle C. Mesenteric fat as a source of C reactive protein and as a target for bacterial translocation in Crohn's disease. Gut. (2012) 61:78–85. 10.1136/gutjnl-2011-300370 21940721 PMC3230831

[13] Goncalves P Magro F Martel F. Metabolic inflammation in inflammatory bowel disease: crosstalk between adipose tissue and bowel. Inflamm Bowel Dis. (2015) 21:453–67. 10.1097/MIB.0000000000000209 25248003

[14] Nell S Suerbaum S Josenhans C. The impact of the microbiota on the pathogenesis of IBD: lessons from mouse infection models. Nat Rev Microbiol. (2010) 8:564–77. 10.1038/nrmicro2403 20622892

[15] Delneste Y Beauvillain C Jeannin P. [Innate immunity: structure and function of TLRs]. Med Sci. (2007) 23:67–73. 10.1051/medsci/200723167 17212934

[16] Kawai T Akira S. TLR signaling. Cell Death Differ. (2006) 13:816–25. 10.1038/sj.cdd.4401850 16410796

[17] Kawasaki T Kawai T. Toll-like receptor signaling pathways. Front Immunol. (2014) 5:461. 10.3389/fimmu.2014.00461 25309543 PMC4174766

[18] Dziarski R Gupta D. Role of MD-2 in TLR2- and TLR4-mediated recognition of Gram-negative and Gram-positive bacteria and activation of chemokine genes. J Endotoxin Res. (2000) 6:401–5. 10.1177/09680519000060050101 11521063

[19] Park BS Song DH Kim HM Choi BS Lee H Lee JO. The structural basis of lipopolysaccharide recognition by the TLR4-MD-2 complex. Nature. (2009) 458:1191–5. 10.1038/nature07830 19252480

[20] Murad S. Toll-like receptor 4 in inflammation and angiogenesis: a double-edged sword. Front Immunol. (2014) 5:313. 10.3389/fimmu.2014.00313 25071774 PMC4083339

[21] Alexander JS Chaitanya GV Grisham MB Boktor M. Emerging roles of lymphatics in inflammatory bowel disease. Ann N Y Acad Sci USA. (2010) 1207 (Suppl. 1):E75–85. 10.1111/j.1749-6632.2010.05757.x 20961310

[22] McNamee EN Rivera-Nieves J. Defective lymphatics in Crohn's disease: tertiary lymphoid follicles plug the gap. Gastroenterology. (2017) 152:908–10. 10.1053/j.gastro.2017.01.022 28153586 PMC9924356

[23] Neal MD Jia H Eyer B Good M Guerriero CJ Sodhi CP. Discovery and validation of a new class of small molecule Toll-like receptor 4 (TLR4) inhibitors. PLoS ONE. (2013) 8:e65779. 10.1371/journal.pone.0065779 23776545 PMC3680486

[24] Agus A Denizot J Thévenot J Martinez-Medina M Massier S Sauvanet P. Western diet induces a shift in microbiota composition enhancing susceptibility to Adherent-Invasive E. coli infection and intestinal inflammation Sci Rep. (2016) 6:19032. 10.1038/srep19032 26742586 PMC4705701

[25] Renshaw M Rockwell J Engleman C Gewirtz A Katz J Sambhara S. Cutting edge: impaired Toll-like receptor expression and function in aging. J Immunol. (2002) 169:4697–701. 10.4049/jimmunol.169.9.4697 12391175

[26] Hirakawa S Hong Y-K Harvey N Schacht V Matsuda K Libermann T. Identification of vascular lineage-specific genes by transcriptional profiling of isolated blood vascular and lymphatic endothelial cells. Am J Pathol. (2003) 162:575–86. 10.1016/S0002-9440(10)63851-5 12547715 PMC1851142

[27] Miyagaki T Sugaya M Okochi H Asano Y Tada Y Kadono T. Blocking MAPK signaling downregulates CCL21 in lymphatic endothelial cells and impairs contact hypersensitivity responses. J Invest Dermatol. (2011) 131:1927–35. 10.1038/jid.2011.135 21593766

[28] Szebeni B Veres G Dezsõfi A Rusai K Vannay Á Mraz M. Increased expression of Toll-like receptor (TLR) 2 and TLR4 in the colonic mucosa of children with inflammatory bowel disease. Clin Exp Immunol. (2008) 151:34–41. 10.1111/j.1365-2249.2007.03531.x 17991289 PMC2276924

[29] Zheng B Morgan ME van de Kant HJG Garssen J Folkerts G Kraneveld AD. Transcriptional modulation of pattern recognition receptors in acute colitis in mice. Biochim Biophys Acta (BBA) Mol Basis Dis. (2013) 1832:2162–72. 10.1016/j.bbadis.2013.07.004 23851050

[30] Tan Y Zou KF Qian W Chen S Hou XH. Expression and implication of toll-like receptors TLR2, TLR4 and TLR9 in colonic mucosa of patients with ulcerative colitis. J Huazhong Univ Sci Technolog Med Sci. (2014) 34:785–90. 10.1007/s11596-014-1353-6 25318894

[31] Fukata M Chen A Vamadevan AS Cohen J Breglio K Krishnareddy S Toll-like receptor-4 (TLR4) promotes the development of colitis-associated colorectal tumors. Gastroenterology. (2007) 133:1869–81. 10.1053/j.gastro.2007.09.008 18054559 PMC2180834

[32] König J Wells J Cani PD García-Ródenas CL MacDonald T Mercenier A. Human intestinal barrier function in health and disease. Clin Transl Gastroenterol. (2016) 7:e196. 10.1038/ctg.2016.54 27763627 PMC5288588

[33] Midtvedt T Bylund-Fellenius AC The role of intestinal bacteria, bacterial translocation and endotoxin in dextran sodium sulphate-induced colitis in the mouse AU - axelsson, L.-G. Microbial Ecol Health Dis. (1996) 9:225–37. 10.3109/08910609609166463

[34] Chassaing B Aitken JD Malleshappa M Vijay-Kumar M. Dextran sulfate sodium (DSS)-induced colitis in mice. Curr Protoc Immunol. (2014) 104:15–25. 10.1002/0471142735.im1525s104 24510619 PMC3980572

[35] Grainger JR Konkel JE Zangerle-Murray T Shaw TN. Macrophages in gastrointestinal homeostasis and inflammation. Pflugers Arch. (2017) 469:527–39. 10.1007/s00424-017-1958-2 28283748 PMC5362667

[36] Gross M Salame T-M Jung S. Guardians of the gut—murine intestinal macrophages and dendritic cells. Front Immunol. (2015) 6:254–254. 10.3389/fimmu.2015.00254 26082775 PMC4451680

[37] Liao S Cheng G Conner DA Huang Y Kucherlapati RS Munn LL. Impaired lymphatic contraction associated with immunosuppression. Proc Natl Acad Sci USA. (2011) 108:18784. 10.1073/pnas.1116152108 22065738 PMC3219138

[38] Heitman B Irizarry A. Infectious disease causes of lymphadenopathy: localized versus diffuse. Lippincotts Prim Care Pract. (1999) 3:19–38. 10214200

[39] Jang MH Sougawa N Tanaka T Hirata T Hiroi T Tohya K. CCR7 is critically important for migration of dendritic cells in intestinal lamina propria to mesenteric lymph nodes. J Immunol. (2006) 176:803–10. 10.4049/jimmunol.176.2.803 16393963

[40] Seth S Oberdörfer L Hyde R Hoff K Thies V Worbs T. CCR7 essentially contributes to the homing of plasmacytoid dendritic cells to lymph nodes under steady-state as well as inflammatory conditions. J Immunol. (2011) 186:3364. 10.4049/jimmunol.1002598 21296980

[41] Kumar V Scandella E Danuser R Onder L Nitschke M Fukui Y. Global lymphoid tissue remodeling during a viral infection is orchestrated by a B cell-lymphotoxin-dependent pathway. Blood. (2010) 115:4725–33. 10.1182/blood-2009-10-250118 20185585

[42] Gregory JL Walter A Alexandre YO Hor JL Liu R Ma JZ. Infection programs sustained lymphoid stromal cell responses and shapes lymph node remodeling upon secondary challenge. Cell Rep. (2017) 18:406–18. 10.1016/j.celrep.2016.12.038 28076785

[43] Kang S Lee SP Kim KE Kim HZ Memet S Koh GY. Toll-like receptor 4 in lymphatic endothelial cells contributes to LPS-induced lymphangiogenesis by chemotactic recruitment of macrophages. Blood. (2009) 113:2605–13. 10.1182/blood-2008-07-166934 19098273

[44] Genovese L Brendolan A. Lymphoid tissue mesenchymal stromal cells in development and tissue remodeling. Stem Cells Int. (2016) 2016:8419104. 10.1155/2016/8419104 27190524 PMC4846763

[45] Takeuchi O Akira S. Pattern recognition receptors and inflammation. Cell. (2010) 140:805–20. 10.1016/j.cell.2010.01.022 20303872

[46] Suresh R Mosser DM. Pattern recognition receptors in innate immunity, host defense, and immunopathology. Adv Physiol Edu. (2013) 37:284–91. 10.1152/advan.00058.2013 24292903 PMC4089092

[47] Shen W Li Y Zou Y Cao L Cai X Gong J. Mesenteric adipose tissue alterations in Crohn's disease are associated with the lymphatic system. Inflamm Bowel Dis. (2018) 25:283–93. 10.1093/ibd/izy306 30295909

[48] Bronsart L Nguyen L Habtezion A Contag C. Reactive oxygen species imaging in a mouse model of inflammatory bowel disease. Mol Imaging Biol. (2016) 18:473–8. 10.1007/s11307-016-0934-0 26873653 PMC4927601

[49] Behr MA. The path to Crohn's disease: is mucosal pathology a secondary event?. Inflamm Bowel Dis. (2010) 16:896–902. 10.1002/ibd.21171 19924803

[50] Laroui H Ingersoll SA Liu HC Baker MT Ayyadurai S Charania MA. Dextran sodium sulfate (DSS) induces colitis in mice by forming nano-lipocomplexes with medium-chain-length fatty acids in the colon. PLoS ONE. (2012) 7:e32084. 10.1371/journal.pone.0032084 22427817 PMC3302894

[51] Brass DM Savov JD Gavett SH Haykal-Coates N Schwartz DA. Subchronic endotoxin inhalation causes persistent airway disease. Am J Physiol Lung Cell Mol Physiol. (2003) 285:L755–L761. 10.1152/ajplung.00001.2003 12794002

[52] Brass DM Hollingsworth JW Cinque M Li Z Potts E Toloza E. Chronic LPS inhalation causes emphysema-like changes in mouse lung that are associated with apoptosis. Am J Respir Cell Mol Biol. (2008) 39:584–90. 10.1165/rcmb.2007-0448OC 18539952 PMC2574529

[53] Shimshoni E Yablecovitch D Baram L Dotan I Sagi I ECM remodelling in IBD: innocent bystander or partner in crime? The emerging role of extracellular molecular events in sustaining intestinal inflammation Gut. (2015) 64:367–72. 10.1136/gutjnl-2014-308048 25416065 PMC4345769

[54] Lu Y Li X Liu S Zhang Y Zhang D. Toll-like receptors and inflammatory bowel disease. Front Immunol. (2018) 9:72. 10.3389/fimmu.2018.00072 29441063 PMC5797585

[55] Midwood K Sacre S Piccinini AM Inglis J Trebaul A Chan E. Tenascin-C is an endogenous activator of Toll-like receptor 4 that is essential for maintaining inflammation in arthritic joint disease. Nat Med. (2009) 15:774–80. 10.1038/nm.1987 19561617

[56] Kim S Kim SY Pribis JP Lotze M Mollen KP Shapiro R. Signaling of high mobility group box 1 (HMGB1) through toll-like receptor 4 in macrophages requires CD14. Mol Med. (2013) 19:88–98. 10.2119/molmed.2011.00001 23508573 PMC3667211

[57] Ehrchen JM Sunderkotter C Foell D Vogl T Roth J. The endogenous Toll-like receptor 4 agonist S100A8/S100A9 (calprotectin) as innate amplifier of infection, autoimmunity, and cancer. J Leukoc Biol. (2009) 86:557–66. 10.1189/jlb.1008647 19451397

